# IRS1 Expression in Hepatic Tissue and Leukocytes in Chronic Hepatitis C Virus Infected Patients: A Comparative Study

**DOI:** 10.1155/2012/698905

**Published:** 2012-07-05

**Authors:** Camila Ripoll Kappel, Nélson A. Kretzmann, Mário Reis Álvares-da-Silva

**Affiliations:** ^1^Gastroenterology and Hepatology Post-Graduate Course, School of Medicine, Universidade Federal do Rio Grande do Sul, Porto Alegre, Brazil; ^2^Experimental Research Center, Hospital de Clínicas de Porto Alegre, Porto Alegre, Brazil; ^3^Gastroenterology Division, Hospital de Clínicas de Porto Alegre, Porto Alegre, Brazil

## Abstract

*Aims.* To determine lymphocyte IRS (IRS1 cells) in HCV patients, correlating it to liver IRS (IRS 1liver) and HOMA-IR. This study tested the hypothesis that IRS1 cells expression can be used as insulin resistance (IR) marker in HCV-infected patients. IRS1 cells were not studied before in HCV infection. *Materials and Methods.* HCV chronically infected patients, naïve, nonobese, noncirrhotic, and nondiabetic were prospectively included and compared to controls (blood donors). Blood was taken, and leukocytes were separated. IRS1 was determined by real-time PCR. Liver tissue was obtained from transplant donors as controls. *Results.* 41 HCV-positive patients were included, 26 males (60.5%); mean age of 45 (±7.9); 33 (80.5%) from genotype 1. 6 out of 12 controls were males (50%); mean age was 26.7 (±3.2). There was expression of IRS1 in leukocytes. The median IRS1 cells (HCV) were 0.061 (0.004 to 0.469); the median IRS 1liver (HCV) was 0.0003 (0.00002 to 0.0186)—lower than in controls (resp., *P* = 0.005 and *P* = 0.018). HOMA-IR had an inverse correlation with IRS 1liver (*P* = 0.04). There was no correlation between IRS1 liver and IRS1 cells (*P* = 0.930). *Conclusions.* There was expression of IRS1 in leukocytes. IRS1 cells and IRS1 liver were lower in HCV patients than in controls.

## 1. Introduction

Chronic infection with hepatitis C virus (HCV) is an independent risk factor for developing type 2 diabetes mellitus (DM) [1–5]. There is a three- to ten-fold increased risk of HCV infection among diabetic patients in comparison with different control groups [4]; this appears to be linked to the development of insulin resistance (IR) [6].

**Figure 1:**
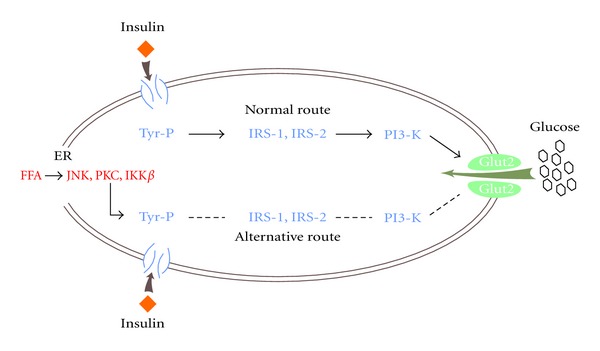
Mechanisms of insulin resistance (adapted from Science Oxcan et al., 2004). The figure shows both normal and alternative route of entry of glucose into the cell. With the increase of nonoxidized fatty acids by the liver, there is a change in the mechanism of tyrosine phosphorylation, and the glucose route of entry into the cell is impaired. Thereafter, intracellular insulin resistance occurs because glucose cannot enter the cell. The insulin resistance is associated with reduced expression of IRS1 and IRS2. These receptors are proteins of a family of ligands and molecules that connect insulin receptors to a cascade of reactions that allow entry of glucose into the cell. FFA: free fatty acids, P-Tyr: tyrosine, PI3-K: phosphatidylinositol kinase, ER: endoplasmic reticulum, JNK: Jun N-terminal kinase, IKK*β*: inhibitor of nuclear factor kappa-b kinase subunit b, PKC: protein kinase C, IRS1 and IRS2: insulin receptor substrates 1 and 2.

The normal route of entry of glucose into the cell involves receptor autophosphorylation and subsequent tyrosine kinase phosphorylation of insulin receptor substrates (IRS1/2) (Figure 1) [7]. The IR may be secondary to alternative serine phosphorylation, as well as due to factors such as obesity, metabolic syndrome, systemic inflammation, and hepatic steatosis, which are sometimes present in patients with HCV [8]. 

High levels of viral RNA and advanced liver fibrosis may be the cause of IR in this population [9]. Moreover, a direct cytopathic effect of HCV on the expression of IRS1 and IRS2 has been suggested [10, 11], particularly in genotype 3 [12]. Indeed, study of pancreatic beta cells and hepatic expression of IRS1 and 2 have shown an increase in hepatic expression of IRS1 and 2 after treatment with interferon and ribavirin [11].

The expression of IRS1 and 2 in lymphocytes has not been demonstrated in humans but has been shown in some animal studies [13, 14]. Impairment of IRS2 expression (but not in IRS1) in monocyte cells was demonstrated in first-degree relatives of type 2 DM patients [15] The objective of this study is to determine the expression of IRS1 in peripheral leukocytes (IRS1 cells) of HCV-infected patients and in a control group (blood donors), correlating it with the expression of IRS1 in the liver (IRS1 liver) and the presence of insulin resistance. Since HOMA-IR is amenable to criticism, and hyperinsulinemic euglycemic clamp, which is the best tool to determine insulin resistance, is difficult to be available in clinical practice, this study tested the hypothesis that IRS1cells expression can be used as IR marker in HCV infected patients. Our objective was to determine lymphocyte IRS (IRS1 cells) in HCV patients, correlating it to liver IRS (IRS1liver) and HOMA-IR.

## 2. Methods

The study included treatment-naïve patients between 18 and 60 years of age with chronic HCV infection (ELISA 3 and HCV-RNA PCR confirmed), regardless of aminotransferase levels and genotype. The exclusion criteria were: patients with clinical, laboratory, and/or biopsy-proven liver disease not associated with HCV, obese (body mass index >30), patients with excessive alcohol consumption (>40 g/day), DM, pancreatic disease, severe cardiovascular disease, chronic renal failure in dialysis therapy, malignant disease, active opportunistic infections, organ transplanted recipients, pregnancy and/or steroids, immunosuppressant or lipid-lowering drugs use. This study was approved by the Ethics Committee of the Hospital de Clinicas de Porto Alegre (HCPA), and all patients signed an informed consent form. The readers of the index tests and reference standard were blinded to the results of the other tests.

After inclusion into the trial, ultrasound-guided liver biopsies were taken from patients, the samples stained with hematoxylin-eosin and then evaluated using the METAVIR score [16]. Patients with stage F0/F1 were grouped as having minimal/mild fibrosis, while those with scores of F2/F3 were rated as having significant fibrosis. Patients with cirrhosis (METAVIR F4) were excluded from the study, as well as those with METAVIR F3 and formation of nodules (classification of Ishak F5) [17].

The rate of fibrosis progression was calculated taking into account the METAVIR score and duration of exposure to the disease, using the formula: fibrosis/duration of disease exposure in years.

The degree of insulin resistance was calculated according to HOMA-IR (Homeostasis Model Assessment-Insulin Resistance), using the measurement of insulin and fasting glucose with the formula: HOMA-IR ={[insulin  (mU/mL) × glucose  (mg/dL)]/405}.

Patients with HCV were divided initially according to genotype and viral load, and then each into two groups, respectively, genotype 3/nongenotype3, and low viral load (<400.000 UI/mL)/high viral load (>400.000 UI/mL).

 Blood and hepatic tissue samples were collected in two stages. Upon first consultation, a 12 mL blood sample was collected in a tube without anticoagulant for subsequent centrifugation, serum separation, and storage at −80°C. A liver biopsy was performed at the next consultation and in addition to the sample of liver tissue, 8 mL of blood was collected using an EDTA tube for separation of leukocytes, as per the manufacturer's protocol (Ficoll-Histopaque). The time between the first and second sample collection did not exceed 60 days. The liver fragment obtained was immediately frozen in liquid nitrogen and stored at −80°C. Flow cytometry was used to assess the population of mononuclear cells isolated. The leukocytes isolated and the liver fragments were used to evaluate the mRNA expression of IRS1 by real-time polymerase chain reaction (real-time PCR). RNA extraction was performed in line with manufacturer instructions using the commercial kit RNeasy mini kit (Qiagen) and was then quantified and its quality tested by photometric measurement. Only high quality RNA was used (A260/A280>1.95). Primers for amplification were obtained from the Harvard Medical School Primer Bank (http://pga.mgh.harvard.edu/primerbank/). The sequences used were as follows: for the IRS-1, forward primer 5′-CTATCCAGCGTACTCCAAAG-3′ and reverse primer 5′-ACAAGTCTGAATGCTCCACT-3′; for relative quantification the beta-microglobulin gene was used. The cDNA synthesis was conducted using the SuperScript III First-Strand Synthesis SuperMix kit in accordance with manufacturer instructions (Invitrogen).

 The PCRs were performed in a final volume of 25 mL, containing 1 *μ*M of both primers, 1x Syber Green Supermix (AppliedBiosystems), and varying amounts of RT product. Amplification was carried out using the Mx3000P real-time PCR Stratagene (GE) system, with data being processed by the fully integrated MX PRO software, using the formula 2^ΔΔCt^. The program profile used for amplification of the gene IRS1 was 95°C for 2 min followed by 45 cycles of denaturation at 95°C for 30 s, annealing at 52°C for 15 s and extension at 60°C for 30 s.

### 2.1. Purification and Sequencing of Amplicons

 The amplicons were purified using the enzymes Exonuclease I (Exo I) and Shrimp Alkaline Phosphatase (SAP) from GE Healthcare. After verification in agarose, 3.33 U of each enzyme was added to 6 uL of the PCR product. Subsequently, the reaction was heated to 37°C for 30 minutes and 80°C for 15 minutes.

 The purified PCR product was then quantified in 1.5% agarose gel for comparison using a Low Mass Reader (Invitrogen). Between 45 and 60 ng, DNA was used for sequencing, together with 1 uL of forward primer and 6 uL water qs. The same primer was used as for the aforementioned PCR, at a concentration of 4 pM.

 The sequencing of amplicons was performed with an automated sequencer ABI 310 Genetic Analyser using the reagent BigDye Terminator v3.1 (Applied Biosystems).

### 2.2. Analysis of Sequencing

 The sequences were analyzed by the Chromas Lite (Technelysium Pty Ltd) program to detect and identify the presence of the same fragment amplification of cDNA samples obtained from the liver and leukocytes. Sequences were compared with the reference sequence PrimerBank ID 5031805a1, (http://pga.mgh.harvard.edu/cgi-bin/primerbank/displayDetail.cgi?primerID=5031805a1) available through Nucleotide BLAST (http://blast.ncbi.nlm.nih.gov/). All sequences were confirmed by reverse sequencing of the tape. 

 Blood samples were collected after 12 hours fasting to determine blood glucose, adiponectin, insulin, and lipid profile (total cholesterol, HDL cholesterol, and triglycerides). Measures of glucose and lipid profile were performed using UV enzymatic hexokinase with the ADVIA 1800 (Siemens). The plasma insulin was measured by electrochemical luminescence using the Roche Modular equipment, and adiponectin was determined according to the ELISA kit protocol (Adiponectin Human ELISA Kit, Biosource).

### 2.3. Statistical Methods

 Considering a standard deviation of 100 [18], a significance level of 0.05, and power of 80%, the sample size necessary in order to detect a difference of 120 units of liver IRS between the HCV and control group was estimated to be at least 27 subjects (9 controls and 18 patients with HCV).

 The data is expressed as a mean ± SD for variables with normal distribution. The median and percentiles of 25 and 75 were used for variables with skewed distribution. The Mann-Whitney test and Spearman's correlation were used for analysis of the results for expression of IRS1. The level of significance adopted was 5%.

 This study was conducted at the research center of the HCPA. Financial support was provided by FIPE (Fundo de Incentivo à Pesquisa e Eventos) of the HCPA. 

## 3. Results

From 93 potentially eligible HCV patients, 41 were included: 26 were male (60.5%); mean age of 45 (±7.9); 33 (80.5%) were genotype 1; 1 (2.4%) was genotype 2; 4 (9.8%) were genotype 3. Of the control group 6 were male (50%) with a mean age of 26.7 (±3.2), lower than that of patients with HCV (*P* < 0.001). Among the 52 patients excluded, 11 were obese (BMI > 30), 2 had cancer, 15 were cirrhotic, 5 had DM, 1 had hepatitis B virus, and 3 had excessive alcohol consumption (>40 g/day).

Table 1 lists the data on glycemic and lipid profiles for patients with HCV in the study. When comparing the results with the METAVIR fibrosis scores, the median insulin was found to be significantly higher in patients with significant fibrosis in contrast to those with minimal/mild fibrosis. Similarly, the HOMA-IR was higher in the group with more advanced fibrosis, but without reaching statistical significance. In relation to high or low viral load, fasting glucose was significantly higher in patients with high viral load.

Using the technique of flow cytometry, a population of mononuclear cells was identified in 83.6% of the total cells, of which 73.4% were CD3 lymphocytes. Sequencing of the product of PCR was carried out to confirm the expression of IRS1 both in leukocytes and in liver tissue and revealed total similarity between the sequences amplified with the cDNA sequence of the IRS1 gene. Expression of IRS1 was seen in leukocytes in both groups. The median IRS1 cells in patients were 0.061 (0.004 to 0.469), significantly lower when compared to controls (*P* = 0.005). The median for expression of IRS1 liver 0.0003 (0.00002 to 0.0186) was also lower in patients (*P* = 0.018).

Table 2 shows the results of IRS1 and its correlation with age, insulin resistance (HOMA-IR), viral load, and rate of progression of fibrosis in patients with hepatitis virus C. There was an inverse association between age and IRS1 cells, while IRS1liver correlated inversely with HOMA-IR. There was no correlation between IRS1 cells and IRS1 liver (Spearman test rho = 0.018 and *P* = 0.930).

## 4. Discussion

 Although the target organ of infection by the hepatitis C virus is the liver, the action of this virus becomes increasingly clear in other organs. Hepatitis C causes more a systemic disease than just a liver disease. Greater attention has recently been given to metabolic disorders caused by HCV, firstly the association with hepatic steatosis and changes in lipid metabolism [19], and more lately its correlation with insulin resistance and DM [1–3, 6], and also cardiovascular risk [20–22]. Hepatitis C can induce a chronic inflammatory state, and inflammatory cytokines are related to decreased expression of IRS1 in nonalcoholic fatty liver disease [3, 18, 23–25]. Indeed, a recently published study from our group [20] demonstrates that HCV patients have increased levels of proinflammatory cytokines (TNF-*α* and IL-6) and also a higher pro-antinflammatory cytokine rate than controls. This is an important finding since some studies have shown that the largest proportion of cellular infiltrate found in a liver with chronic hepatitis C is TH1 cells, such as IL-1b, IL-2, IL-6, IL-8, TNF-*α* and IFN [26]. On the other hand, a recent meta-analysis has demonstrated that sustained virological response is lower in patients with higher HOMA (>2) [27].

 HCV-induced insulin resistance is considered to be more peripheral than hepatic shown by Milner et al. [28] in 29 nonobese infected patients. By means of (1)H-magnetic resonance spectroscopy, authors concluded that HCV induces predominantly muscle insulin resistance, closely related to inflammation, independently of visceral obesity and liver fat.

 In order to assess the direct role of HCV on the mechanisms of action of insulin in different areas (leukocytes and liver tissue), we studied a selected group of patients with chronic hepatitis C infection. The sample selection is one of the strengths of this study as the role of the virus could be better evaluated in a population without obesity, metabolic syndrome, DM, cirrhosis, and with no history of previous antiviral treatment.

 To verify the quality of test, the expression of IRS1 in real-time PCR was confirmed by sequencing of the amplified fragment, based on the cDNA sequence, demonstrating that the amplified fragment in the leukocytes is the same amplified fragment in the liver. The expression of IRS1 in the leukocytes could facilitate the search for surrogate markers of IR in evaluating the prognosis of patients with HCV.

 There is evidence that HCV itself can induce insulin resistance through changes in the route of insulin signaling and the entry of glucose into the cell [1, 9, 18, 29, 30]. This hypothesis is confirmed in this study by demonstrating the decrease of IRS1 in patients with HCV as compared with the control group. The core HCV proteins have a direct cytopathic action on the insulin signaling pathway by decreasing the expression of IRS1, altering the normal route of tyrosine phosphorylation, and entry of glucose into the cell.

 The decreased expression of IRS that seems to be downregulated by the HCV is an important step in the development of IR. Studies have shown that genotype 1 is strongly associated with increased insulin resistance [9, 31], but there are evidences of IR in hepatitis C patients with any genotypes [9]. Most of the included patients in the present study were infected by HCV genotype 1. In the present study, when considered the whole population, patients had no increase in HOMA-IR. Only patients with significant fibrosis had an abnormal test. This is the opposite of the results published by Vanni et al. In their study, also focusing lean and nondiabetic patients, insulin resistance, determined by clamp, was not related to liver fibrosis [18]. On the other hand, in our study, IRS1 was decreased in the whole population and was not influenced by the severity of fibrosis. These findings may suggest that IRS1 decrease is an initial event in this process. An inverse correlation between the expression of IRS1 liver and HOMA-IR was shown in our study, and this is in agreement with the findings demonstrated in liver tissue by Kawaguchi et al. [11]. This study also showed that patients who achieved a sustained virologic response to treatment displayed a decrease in HOMA-IR and HOMA-*β*, in addition to an increased expression of IRS1 and 2 in the hepatocytes. In agreement with these results, Huang et al. recently showed that sustained virological response can improve glucose abnormalities in prediabetic patients with chronic HCV infection [32].

Two studies in animals have demonstrated the expression of IRS1 in leukocytes [13, 14] the first, using a one dog and one cat, evaluated by PCR the expression of IRS1 and IRS2, PI3-K, P-85*α* in different tissues responsive to insulin and in peripheral leukocytes. The aim was to investigate differences in expression of markers found in both species. Authors showed that there is a significant decrease in expression of IRS1, IRS2, PI3-K, P-85*α* in cats as compared to dogs, but they did not address the difference between these markers in tissues like liver, muscle, adipocytes, and leukocytes. In our study, IRS1 both in cells and in the liver was lower in HCV patients than controls, but there was no correlation between the different sites. The second study conducted by the same group cited above used nine dogs when investigating how much an intensive treatment controlling blood glucose levels could cause changes in peripheral leukocytes. Using the same markers as evaluated in the first study, it showed there was a significant increase in IRS1, IRS2, PI3-K, P-85*α* after treatment, and thus the observed changes in peripheral leukocytes were regarded as an improvement in glucose metabolism after treatment with insulin. In addition, the leukocytes were considered sufficiently sensitive enough to monitor the improvement of glycemic control during the intensive treatment therapy of insulin in dogs with DM. Authors considered that insulin has a strong regulatory function in leukocytes and emphasized that these cells are far more accessible and easier to obtain than other insulin sensitive tissues. Our study found decreased expression of IRS1 in both liver tissue and in isolated leukocytes. This is found to be unprecedented in HCV patients and opens a new perspective in relation to the evaluation of insulin resistance. Accessibility to a test that can feasibly use peripheral blood in comparison to the use of a liver biopsy encourages us to develop new tools to assist in the monitoring of these patients. Perhaps, it would be better in the future to compare IRS1cells to hyperinsulinemic-euglycemic clamp in order to really establish its utility as a surrogate marker of insulin resistance.

In conclusion, selected treatment-naïve, nonobese, noncirrhotic, nondiabetic patients with HCV presented a lower expression of IRS1 both in leukocytes and in the liver, and this may be related to the commencement of insulin resistance. IRS1 is inversely correlated with HOMA-IR, which in turn is increased in patients with advanced fibrosis. This study demonstrated the expression of IRS1 in HCV patients for the first time. Further studies are needed to determine whether the leukocyte expression of IRS1 may be used as a substitute marker for liver tissue expression.

## Figures and Tables

**Table 1:** Glycemic and lipid profiles of patients with chronic hepatitis C and its correlation with viral load and fibrosis.

Variable	General	F0/F1	F2/F3	*P*	VL <p400000 UI/mL	VL >400000 UI/mL	*P*
BMI	24.95	24.46	25.57	0.296	23.79	25.03	0.270
(mean ± SD)	(±2.77)	(±2.64)	(±2.86)		(±2.18)	(±2.75)	
Blood Glucose	96.34	94.42	99.64	0.162	88.50	99	0.001
(mean ± SD)	(±11.05)	(±10.64)	(±11.34)		(±4.47)	(±11.72)	
Insulin	9.76	8.16	13.64	0.043	7.68	11.50	0.117
(median 25th–75th%)	(5.83–14.76)	(4.96–12.05)	(7.53–17.03)		(4.74–10.24)	(6.32–16.40)	
HOMA-IR	2.25	1.94	3.51	0.054	1.68	2.76	0.216
(median 25th–75th%)	(1.40–3.78)	(1.17–3.00)	(1.70–4.62)		(1.01–2.44)	(1.50–4.09)	
Total cholesterol	169.28	174.89	161.08	0.211	160	169.38	0.576
(mean ± SD)	(±33.29)	(±38.68)	(±22.29)		(±57.60)	(±27.68)	
HDL cholesterol	52.53	54.21	50.08	0.388	51.33	53.43	0.705
(mean ± SD)	(±13.06)	(±9.60)	(±17.07)		(±9.37)	(±12.35)	
Triglycerides	99	108	96.50	0.855	89	99	0.226
(median 25–75th%)	(70–123)	(69–125.5)	(78.50–129)		(56–108)	(70–123)	
Adiponectin	7.89	6.85	11.86	0.393	9.76	7.89	0.893
(median 25–75th%)	(3.85–17)	(3.03–16.87)	(4.92–17.02)		(3.22–15.61)	(3.23–19.48)	

BMI: body mass index; VL: viral load; F0/F1: minimal/mild fibrosis; F2/F3: significant fibrosis.

**Table 2:** IRS1 in leukocytes and liver of patients with hepatitis C virus and their correlation.

Variable	IRS1 leukocytes			IRS1 liver		
	(*n* = 31)			(*n* = 36)		
	Value	Spearman (rho)	*P*	Value	Spearman (rho)	*P*
Age	44.77	−0.368	0.02	44.89	−0.297	0.07
(mean ± SD)	(±8.4)			(±7.9)		
HOMA-IR	2.63	−0.118	0.55	2.13	−0.341	0.04
(median 25th–75th%)	(1.38–3.86)			(1.28–3.90)		
HCV viral load	1119,225	0.291	0.16	807,000	−0.135	0.44
(median 25th–75th%)	(518,280–3107,865)			(310,000–3101,797)		
Fibrosis	F3—2 (6.9%)	−0.022	0.91	F3—3 (8.1%)	−0.182	0.28
(*n*/%)	F1,2—27 (93.1%)			F1,2—34 (91.9%)		
Fibrosis progression	0,0006876	−0.030	0.87	0,0006876	−0.187	0.28
(median 25th–75th%)	(0.0005–0.00100)			(0.0005–0.00100)		

## Abbreviations

BMI: Body mass index
DM: Diabetes mellitus
HCV: Hepatitis C virus
HOMA-IR: Homeostasis model assessment-insulin resistance
IR: Insulin resistance
IRS: Insulin resistance substrate
PCR: Polymerase chain reaction.
